# Effect of Early Exercise Rehabilitation on Cardiopulmonary Function and Quality of Life in Patients after Coronary Artery Bypass Grafting

**DOI:** 10.1155/2022/4590037

**Published:** 2022-08-10

**Authors:** Rongfang Shan, Lin Zhang, Yuling Zhu, Liyun Ben, Yongli Xin, Fei Wang, Lihua Yan

**Affiliations:** ^1^Department of Nursing, Nantong First People's Hospital, The Second Affiliated Hospital of Nantong University, Nantong, China; ^2^Department of Thoracic and Cardiovascular Surgery, Nantong First People's Hospital, The Second Affiliated Hospital of Nantong University, Nantong, China

## Abstract

The purpose of this study was to analyze the effect of early exercise rehabilitation on cardiopulmonary function and quality of life in patients after coronary artery bypass grafting (CABG). Eighty patients with coronary heart disease who underwent CABG from April 2020 to April 2022 were divided into the study group (*n* = 40) and control group (*n* = 40). The control group was given conventional treatment and routine care after CABG, and the study group received early exercise rehabilitation according to the control group. The cardiac function indexes, 6-minute walking test (6MWT), and cardiopulmonary function indexes and quality of life of the two groups were compared before and after the intervention, and the length of hospitalization and hospital costs as well as the occurrence of pulmonary complications in both groups were recorded. Left ventricular ejection fraction (LVEF) was significantly higher (*P* < 0.05), and left ventricular end-diastolic dimension (LVEDD) and left ventricular end-systolic diameter (LVESD) were significantly lower (*P* < 0.05) in the study group than in the control group after the intervention; 6MWT, maximal oxygen consumption (VO2max), and anaerobic threshold (AT) were significantly higher (*P* < 0.05) in the study group than in the control group after the intervention; physical function (PF), role physical (RP), general health (GH), and role emotional (RE) dimension scores were significantly higher (*P* < 0.05) in the study group compared with the control group after the intervention The differences in the scores of the remaining dimensions were not statistically significant (*P* > 0.05); the total hospitalization time in the test group was significantly shorter than that in the control group (*P* < 0.05), the hospitalization cost was significantly less than that in the control group (*P* < 0.05), and the total incidence of pulmonary infection and hypoxemia was significantly lower than that in the control group (*P* < 0.05). Early exercise rehabilitation can effectively improve cardiopulmonary function and exercise tolerance and improve the quality of life of patients after CABG.

## 1. Introduction

Coronary artery bypass grafting (CABG) is one of the effective ways to treat coronary artery disease, which can significantly relieve patients' myocardial ischemia and hypoxia symptoms and improve their quality of life [1]. In recent years, the prevalence of coronary heart disease has been increasing in China, and the number of patients receiving CABG treatment has been increasing year by year accordingly [2]. As patients may experience loss of cardiopulmonary function, decreased exercise endurance, and negative psychological emotions after CABG, this affects patients' cardiopulmonary rehabilitation and prognostic regression, leading to a decrease in their quality of life [3]. Therefore, cardiac rehabilitation of patients after CABG is crucial. The American Association of Cardiovascular and Pulmonary Rehabilitation (AACVPR) recommends [4] that the different developmental stages of cardiac rehabilitation in coronary artery disease should be divided into three phases, namely, phase I rehabilitation (in-hospital rehabilitation phase), phase II rehabilitation (out-of-hospital early rehabilitation/outpatient rehabilitation phase), and phase III rehabilitation (out-of-hospital long-term rehabilitation phase). Early motor rehabilitation is the core component of phase I rehabilitation for CABG patients, which refers to the patient's stable condition, and motor training can be started 4 hours after surgery [5]. Current studies have confirmed that cardiac rehabilitation with exercise rehabilitation as the core can bring benefits to postoperative patients with CABG [5] but mainly in the phase II and phase III rehabilitation stages. The study of early exercise rehabilitation care for patients with CABG surgery is still at the beginning stage, and more studies have to be conducted to prove it. In this study, we analyzed the effects of early exercise rehabilitation on postoperative cardiopulmonary function and quality of life in patients undergoing CABG surgery, aiming to provide feasible intervention strategies for the rehabilitation of patients after CABG surgery.

## 2. Patients and Methods

### 2.1. Patients

Eighty patients with coronary heart disease who were admitted to the hospital for CABG surgery from April 2020 to April 2022 were selected as the subjects of this study. Inclusion criteria were as follows: (1) all patients met the relevant diagnostic criteria for coronary heart disease [6]; (2) patients were to be treated with CABG surgery; (3) both lower limbs were physically functional; (4) patients were conscious, aware of the process and purpose of this study, and agreed to sign the informed consent form. Exclusion criteria were as follows: (1) patients with other cardiac lesions combined with simultaneous surgical management, such as valvular lesions, macrovascular lesions, and ventricular wall tumors; (2) we excluded emergency CABG, minimally invasive CABG, or recardiac surgery; (3) patients with severe dysfunction of important organs such as the lung, liver, and kidney; (4) patients with severe infectious shock due to recent major surgery or trauma; (5) pregnant or lactating women; and (6) patients with malignant tumors. Patients who met the inclusion and exclusion criteria were divided into study and control groups according to the nonsimultaneous control method, with 40 cases in each group. There was no statistically significant difference in gender, age, New York Heart Association (NYHA) cardiac function class, and smoking status between the two groups (*P* > 0.05) (Table 1). The study was reviewed and approved by Nantong First People's Hospital medical Ethics Committee.

### 2.2. Methods

In the control group, the following measures were taken. Routine postoperative CABG care for patients included admission education, preoperative instructions (preoperative dietary precautions, introduction to the specialty ICU environment), postoperative instructions (postoperative dietary instructions, drainage tubes, recording of in and out volume), and predischarge education.

Early exercise rehabilitation was implemented on the basis of the control group. The specific exercise rehabilitation program was as follows: first, the content of cardiac exercise rehabilitation in ICU inpatients was dominated by pulmonary rehabilitation and exercise. (1) We evaluated the patient's arterial blood gases, chest radiograph, and symptoms and instructed the patient to perform abdominal breathing exercises, 2 times/d for 5–10 min/time, with appropriated adjustment of ventilator parameters, and advised the patient to do so in a state of spontaneous breathing. We promoted pulmonary resuscitation with the help of somatic therapy for lung expansion. (2) Once the patient was out of the acute-danger period during tracheal intubation and the condition was stable and contraindications are excluded, early bed activities could be started. (1) We increased the angle of the patient's bed head so that the patient transitions from a semisitting position to a sitting, independent sitting, and bedside sitting position. Patients underwent joint mobility training and low-intensity resistance training for 5–10 min initially, and the intensity of activity was adjusted according to the patient's heart rate, blood pressure, oxygen saturation, and respiratory rate. (3) Pulmonary rehabilitation exercises were performed after estuation. Patients were assisted in lip retraction breathing training, abdominal breathing training, sputum expulsion training, back patting, and deep breathing, 2 to 3 groups/d, 10 to 15 min/time. (4) Exercise training was performed after the patient was extubated. When the patient's muscle strength was <3, only passive joint exercises were performed, 2 times/d; when the patient's muscle strength was 3–5, the patient was helped to perform postural training, 8–10 times/group, 2–3 groups/d; when the patient's muscle strength reached 5, the patient was helped to perform resistance training, 8–10 times/group, 2–3 groups/d. Once the mean arterial pressure <65 mmHg or >110 mmHg, heart rate <50 beats/min or >130 beats/min, respiratory rate <12 beats/min or >40 beats/min, oxygen saturation <88%, obvious human-machine confrontation, as well as the patient's poor subjective state of feeling and fall, pneumonectomy tube displacement, drainage tube prolapse, etc., after CABG, the patient should promptly suspend the rehabilitation content and inform the physician in charge. Second, the contents of exercise rehabilitation during the postoperative ward are as follows. (1) Lung training: (1) In the 1st d postoperative period, we encouraged patients to perform breathing exercises such as abdominal breathing, lip retraction breathing training, and deep breathing, together with the use of breathing trainers, effective coughing, chest tapping, and breathing exercises until discharge. (2) For patients with sputum retention and pulmonary atelectasis, airway contouring techniques could be performed on the basis of protecting the patient's wound. If the target effect was not achieved, coughing and breathing exercises can be combined with postural management and thoracic tapping to assist coughing and breathing exercises. (3) We performed breathing exercises training for patients. (2) Functional training: In the 1st d after surgery, patients were instructed to perform passive or active limb activities in bed and then gradually transitioned from active limb activities in the bed to bedside activities, walking in the ward, and up and down stairs training every day. Aerobic training: Within the patient's tolerance range, the patient can increase to low-to-medium-intensity aerobic exercise step by step and can choose bedside treadmill training or walking on the ground. The patient's exercise time is increased from 5 minutes to 10–20 minutes, and the patient's symptoms, signs, and electrocardiogram should be closely monitored during the exercise. Shoulder joint training: In the 1st postoperative day, patients can perform appropriate shoulder joint activities such as shoulder lift, shoulder loop, and head wrap-around movements without discomfort, 2 times/d until discharge. Third, discharge assessment. Before discharge, patients' cardiopulmonary function, exercise endurance, and quality of life were assessed, and a short-term postdischarge rehabilitation program was developed for them according to their cardiopulmonary function and exercise endurance.

### 2.3. Evaluation Indicators

Cardiopulmonary function, exercise tolerance, and quality of life were assessed before and after the intervention (before discharge). (1) Cardiac function: left ventricular ejection fraction (LVEF), left ventricular end-diastolic diameter (LVEDD), and left ventricular end-systolic diameter (LVESD) were measured by color Doppler flow imaging (CDFI). (2) 6-minute walk test (6MWT): the patient walked as fast as possible for 6 min in the room along a graduated corridor of 30 m in length, and the total distance the patient walked was recorded. (3) Cardiopulmonary function: cardiopulmonary function was assessed using a cardiopulmonary function test system, and specific parameters included maximum oxygen uptake (VO2max) and anaerobic threshold (AT). (4) Quality of life: the quality of life of patients was assessed using the Chinese version of the Short Form-36 (SF-36) [7], which consists of 36 items and includes 8 dimensions: physical function (PF), physical function (RP), body pain (BP), general health (GH), vitality (VT), social function (SF), emotional role (RE), and mental health (MH), with a full score of 100 points in each dimension, indicating that the patient's quality of life is better. (5) Length of hospital stay and hospitalization costs: ICU length of stay, total length of stay, and hospital costs were recorded. (6) Pulmonary complications: complications such as pulmonary infection and hypoxemia were recorded, and the total incidence was calculated.

### 2.4. Statistical Analysis

The data were processed using the statistical software Statistical Product and Service Solutions (SPSS) 25.0 (IBM, Armonk, NY, USA). Measurement data were expressed as mean ± standard deviation (x̅ ± *s*) Independent-sample and paired-sample *t*-tests were performed for comparisons within groups. Enumeration data were expressed as constituent ratio (%), and the *χ*^2^ test or Fisher's exact probability test was performed for comparison. Differences were considered statistically significant at *P* < 0.05.

## 3. Results

### 3.1. Cardiac Function Indicators before and after Intervention between the Two Groups

Before intervention, there was no significant difference in LVEF, LVEDD, and LVESD between the two groups (*P* > 0.05). After intervention, LVEF in the test group was significantly higher than that in the control group (*P* < 0.05), and LVEDD and LVESD were significantly lower than those in the control group (*P* < 0.05) (Table 2).

### 3.2. 6MVT and Cardiopulmonary Function Parameters after Intervention between the Two Groups

Before intervention, there was no significant difference in 6MVT, VO2max, and AT between the two groups (*P* > 0.05). 6MVT, VO2max, and AT in the test group were significantly higher than those in the control group after intervention (*P* < 0.05) (Table 3).

### 3.3. Quality of Life before and after Intervention between the Two Groups

Before intervention, there was no significant difference in the scores of all dimensions of SF-36 scale between the two groups (*P* > 0.05). After the intervention, compared with the control group, the PF, RP, GH, and RE dimension scores in the test group were significantly increased (*P* < 0.05), and there was no significant difference in the other dimension scores (*P* > 0.05) (Table 4).

### 3.4. Hospital Stay and Hospitalization Costs between the Two Groups

After the intervention, there was no significant difference in ICU length of stay between the two groups (*P* > 0.05). The length of hospital stay was significantly longer in the control group (*P* < 0.05), and the hospitalization costs were significantly less than those in the control group (*P* < 0.05) (Table 5).

### 3.5. The Incidence of Pulmonary Complications after Intervention between the Two Groups

After intervention, the total incidence of pulmonary infection and hypoxemia in the test group was significantly lower than that in the control group (*P* < 0.05) (Table 6).

## 4. Discussion

The invasive nature of the operation in post-CABG patients leads to a restriction of thoracic movement, which affects the gas exchange process and reduces lung function, thus limiting exercise capacity. [8]. Therefore, early intervention after CABG is important. Ohbe et al. [9] showed that early rehabilitation can help to reduce in-hospital mortality, total hospital costs, ICU length of stay, and total hospital stay after CABG.

Cardiac rehabilitation has been widely used in the postoperative management of patients with coronary heart disease [10]. Previous studies [11–13] have shown that cardiac rehabilitation has benefits on physical function, cardiopulmonary function, cardiopulmonary symptoms, and exercise endurance in patients after CABG. However, the benefits of cardiac rehabilitation for patients after CABG are mainly found in the phase II and phase III rehabilitation stages [14]. There is limited evidence to support the benefit of phase I rehabilitation for patients after CABG, and it is unclear how valuable phase I rehabilitation is in patients after CABG. The results of this study showed that LVEF was significantly higher, and LVEDD and LVESD were significantly lower in the study group than in the control group after intervention, indicating that early exercise rehabilitation can effectively improve cardiac function in patients after CABG. This may be related to the addition of pulmonary rehabilitation training and exercise during the trial ICU and in the postoperative ward. Cardiac rehabilitation training can effectively increase the adaptability of cardiac function and improve the coronary blood supply capacity and vascular elasticity, thereby promoting the recovery of cardiac function [15]. However, the relationship between early exercise rehabilitation and LVEF, LVEDD, and LVESD needs to be verified by more clinical trials. 6MVT is commonly used in the efficacy assessment of therapeutic interventions for coronary heart disease and is a valid indicator for testing exercise tolerance in patients with cardiovascular disease [16].

The indicators of cardiopulmonary exercise test were commonly used include VO2max, AT, etc., of which VO2max is an important indicator of cardiopulmonary function and aerobic endurance [17]; AT can reflect the potential of the body to tolerate load [18]. The results of this study showed that 6MWT, VO2max, and AT in study group were significantly higher than those in the control group after intervention, suggesting that early exercise rehabilitation can effectively improve cardiopulmonary function and exercise endurance in patients after CABG.

The reasons for this are that early exercise rehabilitation, led by pulmonary rehabilitation and exercise, is used throughout the cardiac rehabilitation process in the ICU and in the postoperative ward, not only to improve the efficiency of the patient's postoperative pulmonary ventilation but also to enhance the patient's skeletal muscle strength and tolerance, thus improving the patient's exercise tolerance. The other study [19] reported that cardiac rehabilitation guided by the cardiopulmonary exercise test can improve VO2max and AT and exercise tolerance in patients with coronary heart disease after percutaneous coronary intervention, which is an important means to promote postoperative rehabilitation of patients.

Previous studies [20] have suggested that improving the quality of life of patients with cardiovascular disease is one of the important goals of cardiac rehabilitation. The results of this study showed that after the intervention, compared with the control group, the PF, RP, GH, and RE dimension scores in the test group were significantly increased, suggesting that early exercise rehabilitation can effectively alleviate the physical and mental health of patients after CABG and then improve the quality of life of patients. The following reasons were considered: 1. as the cardiopulmonary function of post-CABG patients improved, their physiological health improved; 2. patients' adherence to early rehabilitation exercises increased their exercise endurance, which in turn increased their confidence in overcoming postoperative exercise barriers, alleviated their postoperative fears and improved treatment compliance, which was conducive to accelerating postoperative rehabilitation. The study [21] also showed that phase I exercise rehabilitation could increase 6MVT and improve the quality of life of patients after CABG. In addition, the results of this study showed that after the intervention, the total length of stay in the trial group was significantly shorter than that of the control group, the hospitalization cost was significantly less than that of the control group, and the total incidence of pulmonary infection and hypoxemia was significantly lower than that of the control group. It was suggested that early exercise rehabilitation helped to shorten the total length of stay, reduce hospital costs, and lower the incidence of complications such as pulmonary infections and hypoxemia. Therefore, this study tentatively confirms that early exercise rehabilitation is effective in improving cardiopulmonary function and quality of life in post-CABG patients and that by formulating a reasonable early exercise rehabilitation program for patients, it can help promote early recovery and help them return to society as soon as possible. However, the sample size collected in this study was small, and the observation time was limited. It is necessary to expand the sample size and extend the observation time to further analyze the effect of early exercise rehabilitation for post-CABG patients. This paper has the following limitations: 1. the patients included in this paper exclude patients with minimally invasive CABG and repeat CABG, so the role of these patients still needs further research; 2. the sample size of the study is small; 3. the article fails to carry out. The study was conducted in a randomized double-blind method.

## 5. Conclusion

In conclusion, early exercise rehabilitation can effectively improve cardiopulmonary function and exercise tolerance and enhance the quality of life of patients after CABG. It can provide a feasible intervention strategy for the rehabilitation of post-CABG patients, which is worthy of consideration and has clinical application value.

## Figures and Tables

**Table 1:** Comparison of general data between the two groups.

Group	Number of subjects	Gender (case)		Age (years, *x̅* ± *s*)	NYHA classification (case)			Smoking (case)	
		Male	Female		Grade II	Grade III	Grade IV	Yes	No
Control group	40	29	11	63.58 ± 8.14	25	15	0	10	30
Study group	40	28	12	64.06 ± 7.93	23	16	1	13	27
Χ^2^/*t*-value		0.061		0.231	0.530			0.549	
*P*-value		0.805		0.818	0.596			0.459	

**Table 2:** Comparison of cardiac function parameters before and after intervention between the two groups (*x̅* ± *s*).

Group	Time	LVEF (%)	LVEDD (mm)	LVESD (mm)
*Control group* (*n* = 40)	Preintervention	52.49 ± 7.81	50.27 ± 6.35	47.31 ± 5.79
	Postintervention	58.91 ± 10.03	46.43 ± 5.86	42.15 ± 4.48
	*t*-value	3.194	2.811	4.458
	*P*-value	<0.001	0.001	<0.001

*Study group* (*n* = 40)	Preintervention	53.16 ± 7.58	50.09 ± 6.32	47.22 ± 5.61
	Postintervention	63.04 ± 11.69^*∗*^	43.26 ± 5.07^*∗*^	38.60 ± 4.13^*∗*^
	*t*-value	4.485	5.331	7.826
	*P*-value	<0.001	<0.001	<0.001

Note: Compared with the control group after intervention, *P* < 0.05.

**Table 3:** Comparison of 6MVT and cardiopulmonary function parameters after intervention between the two groups (*x̅* ± *s*).

Group	Time	6MVT (m)	VO2max (mL/kg/min)	AT (mL/kg/min)
*Control group* (*n* = 40)	Preintervention	375.24 ± 18.06	15.39 ± 1.27	10.16 ± 1.24
	Postintervention	407.61 ± 22.49	16.52 ± 1.04	10.84 ± 1.08
	*t*-value	7.098	4.354	2.615
	*P*-value	<0.001	<0.001	0.011

*Study group* (*n* = 40)	Preintervention	372.19 ± 17.67	15.31 ± 1.25	10.22 ± 1.31
	Postintervention	441.83 ± 25.34^*∗*^	17.96 ± 1.09^*∗*^	12.47 ± 1.02^*∗*^
	*t*-value	14.257	10.106	8.571
	*P*-value	<0.001	<0.001	<0.001

Note: Compared with the control group after intervention, *P* < 0.05.

**Table 4:** Comparison of quality of life before and after intervention between the two groups ( x ®± *s* (points)).

Group	Time	PF	RP	BP	GH	VT	SF	RE	MH
*Control group* (*n* = 40)	Preintervention	73.68 ± 5.13	58.41 ± 4.79	59.84 ± 8.46	55.03 ± 6.27	70.66 ± 5.84	81.24 ± 4.72	69.16 ± 7.07	73.72 ± 5.36
	Postintervention	78.43 ± 7.55	62.36 ± 5.17	60.07 ± 9.32	60.29 ± 7.03	71.42 ± 6.30	80.91 ± 4.95	72.84 ± 6.49	74.56 ± 6.18
	*t*-value	3.291	3.545	0.116	3.527	0.560	0.305	2.425	0.649
	*P*-value	0.002	0.001	0.908	0.001	0.577	0.761	0.018	0.518

*Study group* (*n* = 40)	Preintervention	73.61 ± 5.27	58.27 ± 4.63	59.49 ± 8.41	55.11 ± 6.35	70.57 ± 5.89	81.09 ± 4.66	69.23 ± 7.01	73.67 ± 5.44
	Postintervention	81.28 ± 7.96^*∗*^	66.04 ± 5.74^*∗*^	60.11 ± 9.35	71.43 ± 7.29^*∗*^	71.33 ± 6.42	82.15 ± 4.78	77.58 ± 8.26^*∗*^	74.89 ± 6.31
	*t*-value	5.081	6.664	0.312	10.676	0.552	1.004	4.875	0.926
	*P*-value	<0.001	<0.001	0.756	<0.001	0.583	0.318	<0.001	0.357

Note: Compared with the control group after intervention, *P* < 0.05.

**Table 5:** Comparison of hospital stay and hospitalization costs between the two groups (*x̅* ± *s*).

Group	Number of subjects	ICU length of stay (d)	Total hospital stay (d)	Hospital costs (ten thousand yuan)
Control group	40	2.86 ± 1.32	15.74 ± 4.59	12.06 ± 1.43
Study group	40	2.44 ± 1.25	13.26 ± 3.83	9.98 ± 1.69
*t*-value		1.461	2.624	5.942
*P*-value		0.148	0.011	<0.001

**Table 6:** Comparison of incidence of pulmonary complications after intervention between the two groups [*n* (%)].

Group	Number of subjects	Lung infection	Hypoxemia	Total occurrence
Control group	40	5 (12.50)	4 (10.00)	9 (22.50)
Test group	40	1 (2.50)	1 (2.50)	2 (5.00)
Χ^2^-value		—	—	5.165
*P*-value		0.201	0.359	0.023

## Data Availability

The datasets used and analyzed during the current study are available from the corresponding author on reasonable request.
